# Practice Variations in the Diagnosis and Treatment of Pulmonary Embolism

**DOI:** 10.1155/2024/6633148

**Published:** 2024-11-04

**Authors:** Devesh Thakkar, Frances Garden, John Nguyen, Brenda Ta, Sikandar Hussaini, Claudia C. Dobler

**Affiliations:** ^1^Department of Respiratory and Sleep Medicine, Liverpool Hospital, Sydney, Australia; ^2^South West Sydney Clinical Campus, School of Clinical Medicine, University of New South Wales, Sydney, Australia

**Keywords:** clinical variation, evidence-based medicine, pulmonary embolism, venous thromboembolism

## Abstract

Venous thromboembolism is responsible for a significant burden of disease worldwide. Despite the publication of multiple international guidelines, anecdotal evidence suggests significant clinical variation exists in the diagnostic and management pathways of pulmonary embolism (PE). We conducted a retrospective cohort study using electronic medical records to examine clinical variation in patients admitted to a tertiary referral center in Australia with a diagnosis of PE between November 2018 and January 2020. Three hundred cases met the inclusion criteria; we found variation in rates of compression ultrasonography, acute investigation of the right ventricle, and planning of repeat imaging at specialist follow-up. Guidelines do not address the use of compression ultrasonography in already diagnosed PE, are conflicting in their recommendation for acute investigation of the right ventricle, and recommend repeat imaging only if there are persistent symptoms at the time of specialist follow-up. The variations we found in this study may in part be due to physician preference or due to the paucity of evidence for some of these diagnostic practices. Robust future studies are required to guide the use of these investigations in PE.

## 1. Introduction

Venous thromboembolism (VTE) is responsible for a significant burden of disease with estimated rates of 1–2/1000 person-years in the United States [1, 2]. Up to one-third of patients will have recurrence of VTE within 10 years after an initial episode, indicating the need for long-term follow-up and management of patients [1]. Within the subset of patients with VTE, those who have pulmonary embolism (PE) are at high risk of hemodynamic compromise during the acute phase, often necessitating hospital admission and intensive monitoring. Furthermore, up to half of patients with PE report limitations of function or quality of life many years after an event, with 10%–30% of patients exhibiting abnormal pulmonary artery pressure and up to 4% being diagnosed with chronic thromboembolic pulmonary hypertension (CTEPH). Together, these manifestations form the complex post-PE syndrome [3].

Diagnostic pathways for PE are complex, as PE symptoms overlap with symptoms of other common diagnoses, including acute coronary syndrome, respiratory infections, and acute exacerbations of chronic respiratory diseases. Additionally, diseases such as chronic obstructive pulmonary disease increase the risk of developing PE, adding to the difficulty of diagnosis [4]. Many patients are diagnosed with PE during investigation for other pathologies, particularly on progress imaging for patients with malignancy [5]. A diagnosis of PE is made by computed tomography pulmonary angiogram (CTPA) or ventilation-perfusion (V-Q) imaging. Low diagnostic yields, in particular with CTPAs, emphasize the importance of assessing the pretest probability for PE before progressing to a diagnostic imaging test [6]. There are several risk prediction tools available for patients presenting to the Emergency Department (ED) such as the Wells score or modified Geneva score; however, uptake of these tools is generally poor [7, 8]. Once a diagnosis of PE has been made, ancillary tests may be performed to guide further management, including transthoracic echocardiogram (TTE), blood troponin levels, and lower limb compression ultrasonography (CUS). PE (and VTE in general) is treated with anticoagulation, the duration of which is determined by transient risk factors present at the time of diagnosis.

The European Society of Cardiology (ESC) in association with the European Respiratory Society (ERS) [9], the American Society of Haematology (ASH) [10], the Thrombosis and Haemostasis Society of Australia and New Zealand (THANZ) [11], and the CHEST group [12] have published comprehensive guidelines on the diagnosis and management of PE. However, the evidence underpinning a number of these guideline recommendations is poor due to the lack of randomized controlled trials.

Our anecdotal experience suggests that there is significant clinical variation in the diagnosis and treatment of PE, influenced by individual physician preference, possibly because of a lack of strong evidence underpinning some recommendations. Clinical variation is a difference in healthcare processes or outcomes when compared to peers or to a gold standard, such as an evidence-based guideline recommendation [13]. Studies have shown that variation from guidelines issued by major international medical societies is common in clinical practice [14, 15].

Unwarranted clinical variation, defined as “variations that cannot be explained by illness characteristics or patient preference” [16], can be associated with poor health outcomes and unnecessary cost. In this study, we aimed to assess clinical variation, both at an individual clinician and specialty-group level, in the diagnostic and treatment pathways of PE. We further aimed to examine whether there were guideline recommendations for areas of variation (possibly indicating “unwarranted variation”), and, if so, what the strength of the evidence underpinning the guideline recommendation was.

## 2. Methods

The study was approved by the Southwestern Sydney Local Health District (SWSLHD) Human Research Ethics Committee (project identifier: 2019/ETH13076).

### 2.1. Study Setting and Participants

We conducted a retrospective cohort study using electronic medical records of patients discharged from Liverpool Hospital, a tertiary-level public hospital in Sydney, Australia, between November 2018 and January 2020 with a discharge diagnosis of PE. Patients were excluded if the initial diagnostic investigation was performed outside of Liverpool Hospital. Patients who had multiple admissions for the same episode of PE were only included once in the analysis.

### 2.2. Data Collection

Data were collected on patient characteristics, on medical information relevant to the diagnosis and management of PE, and on hospital admission details including the length of stay. Medical information collected included presenting complaint; location of initial PE-focused investigation (ED, Intensive Care Unit [ICU] or ward); risk factors which were likely to affect choice or duration of treatment; investigations, including both diagnostic (CTPA, other CT chest, or V-Q imaging) and ancillary investigations (troponin, TTE, CUS); initial and long-term treatment plan; and clinical and/or imaging follow-up plan. Only data directly relevant to the diagnosis of PE were collected; for example, tests such as arterial blood gases were not collected as these are not useful in the diagnosis of PE. Furthermore, our institution utilizes troponin as the primary blood investigation of cardiac dysfunction rather than b-type natriuretic peptide (BNP), and so, this was collected as primary blood investigation of the right ventricle. A list of collected parameters can be found in Appendix A.

### 2.3. Comparative Groups (Treating Physician)

In the study setting, patients admitted to hospital with PE are usually managed by respiratory physicians, whereas patients who are diagnosed with PE during their hospital stay generally remain under the admitting physician who may elect to ask for advice from a consulting respiratory physician.

For this study, the main attending physician at hospital discharge was considered the treating physician. We grouped patients as follows: Group A consisted of patients under the care of a respiratory physician, Group B included patients primarily treated by a nonrespiratory physician for which respiratory specialist input was sought for the diagnosis and/or management of PE, and Group C included patients with PE who were managed by a nonrespiratory physician without respiratory specialist input. A combined group was formed from Group A and Group B (Group A + B) including all patients in whom respiratory specialist input with regard to PE diagnosis and/or treatment was present.

### 2.4. Statistical Analysis

Statistical analysis was performed in SAS version 9.4. Chi-square tests were used to examine associations between diagnostic pathways and investigations, the location of initial suspicion for PE (ED vs. ICU/ward), and treating physician's specialty. We also evaluated variation in diagnostic procedures and treatments between individual physicians by comparing the percentage of procedures and treatments ordered by each individual physician. Chi-square tests were also used to examine the association between management and follow-up treatment plans and treating physician team's specialty. We explored recommendations from international guidelines, namely the ESC/ERS and ASH guidelines for the diagnostic tests and treatments that were prescribed [10, 11].

## 3. Results

A total of 300 patients with a mean age of 62 years (standard deviation [*SD*] = 17.4), of which 159 (53%) were male, met the inclusion criteria during the study period. Patient characteristics are shown in Table 1.

### 3.1. Presentation and Diagnostic Investigations

Diagnostic pathway data are presented in Table 2. ED presentations accounted for just over one-half of all cases (*n* = 161; 54%). Wells' score was documented in only 16 of these cases (10%). The initial investigation was CTPA in almost half of all cases (*n* = 148; 49%). D-dimer was utilized as the initial investigation in 34 (11%) patients; except for one, all of these were patients presenting to ED. There was a significant difference in the choice of the initial investigation type between patients from ED and ward/ICU patients (*p* < 0.0001). Most patients (*n* = 233; 78%) had at least one risk factor for VTE, the most common of which was active malignancy (*n* = 100; 33%).

The majority of PE were diagnosed on CTPA (*n* = 199; 66%); 65 of these were classified as proximal PE (main or lobar pulmonary artery). Of the 60 PEs diagnosed on V-Q scan, all but three were classified as distal PE.

PE was diagnosed incidentally in 41 (14%) patients on CT imaging performed for other reasons. The proportion of pulmonary emboli diagnosed on incidental CT scanning was higher in the inpatient population (including ICU) compared to ED (24% vs. 4%).

### 3.2. Ancillary Investigations

Blood troponin levels were performed at diagnosis in just over half of patients (*n* = 167; 56%) and were elevated in 93 patients (56% of tests). They were more likely to be performed in Group A + B than Group C (65% vs. 36%, *p* < 0.0001). In patients who were diagnosed on CTPA, troponins were performed in all patients with proximal PE (main or lobar pulmonary artery), but only 43% of patients with distal PE (segmental or subsegmental PE). A TTE was performed in 82 patients (27%); 51 of these patients were diagnosed on CTPA. Just over one-third of patients (*n* = 103) did not have dedicated investigation of the right ventricle (neither TTE nor troponin test); this was 25% of patients in the A + B group, but 51% of the remaining patients. CUS was performed in 132 patients (44%) and was more likely to be requested by Group A + B than Group C (49% vs. 33%, *p* = 0.01). Of those who had a CUS, 69 of these had confirmed DVT (52% positive), while four patients had CUS outside the hospital following discharge, the results of which were unknown. When examining Group A, there was variation between individual respiratory specialists in the rates of ordering CUS (14%–62%). A hereditary and acquired thrombophilia screen was ordered and performed in 43 patients (14%).

### 3.3. Management and Follow-Up

Management pathway data are presented in Table 3. The median total length of stay was 8 days overall, but only 4 days in patients diagnosed in ED (in whom PE was likely the reason for admission) compared to 18 days in patients already admitted to the ward/ICU at the time of diagnosis (who were admitted for reasons other than PE).

Thrombolysis was given in nine (3%) patients. The initial anticoagulation choice was low molecular weight heparin in 138 patients (46%), unfractionated heparin in 99 patients (33%), and direct-acting oral anticoagulants in 52 patients (17%); warfarin was continued in four patients (1%) who were already anticoagulated. The remaining seven patients were not anticoagulated after diagnosis. Direct-acting oral anticoagulants were the most common choice for maintenance anticoagulation (*n* = 162; 54%), followed by warfarin (*n* = 63; 21%) and low molecular weight heparin (*n* = 51; 17%). Twenty-four patients were not anticoagulated at discharge (8%). Nineteen of these patients had one or more documented reasons for withholding anticoagulation, with nine patients deemed palliative, six patients with bleeding risk, three with documented “small PE,” three with documented uncertainty as to the diagnosis, one with an undisclosed adverse effect, and one patient who declined treatment. There was no difference between Group A + B and Group C as to the choice of agent for initial anticoagulation (*p* = 0.17); however, there was a difference in the maintenance choice of anticoagulation (*p* < 0.0001).

An inferior vena cava filter was utilized in 21 patients (7%); 16 of these had CUS performed, of which 13 had confirmed lower limb DVT. Twenty patients were documented to have a high bleeding risk; 12 of these had CUS. Four of these 20 patients had IVC filters inserted (three of these had CUS performed; all three had confirmed DVT).

Follow-up imaging was planned at discharge in 36 patients (12%); the majority of these (*n* = 33, 92%) were in the A + B group. In three of these patients, there was some indication of uncertainty of diagnosis. The modality of choice for reimaging was usually the same as the diagnostic imaging modality for PE. A plan for reimaging at follow-up was more likely in Group A + B compared to Group C (*p* = 0.0012). There was a large variation noted between individual respiratory physicians within the A + B group (0%–50% of each physician's respective patients were planned for reimaging during follow-up).

## 4. Discussion

Our retrospective cohort study demonstrated differences in the utilization of CUS, investigation of the right ventricle with blood troponin test or TTE, and the planning of follow-up reimaging between PE-treating teams that contain a respiratory consultant compared to a team that does not. A summary of the relevant recommendations from guidelines is presented in Table 4.

### 4.1. CUS (Lower Limb Venous Doppler Examination)

Our results showed differences between individual physicians and a significant difference between the A + B group and Group C with regard to ordering of CUS. CUS is a widely available and radiation-free tool used to diagnose deep vein thrombosis (DVT) of the lower limb, commonly thought to be the cause of PE, as well as a diagnosis in its own right. When evaluating possible PE, ESC [9] and ASH guidelines [11] both recommend the use of CUS in patients in whom primary imaging of the chest is associated with an increased risk of adverse events (for example, pregnant women). If DVT is diagnosed, then confirmation of a diagnosis of PE is unnecessary in most patients, as the anticoagulation treatment is the same for VTE. In our study, CUS was utilized as the initial investigation in only seven patients, all of whom went on to have CTPA or V-Q scanning to confirm the diagnosis. One hundred twenty-five patients underwent CUS following the diagnosis of PE; no recommendation is made within the guidelines for the use of CUS in this manner [9–12].

Some data suggests the presence of DVT at the time of PE diagnosis is an independent risk factor for mortality following a diagnosis of PE [17, 18], although other studies failed to find a statistically significant correlation between presence of DVT and unfavorable outcome [19]. More research is required to determine whether CUS should be used for risk stratification in confirmed PE to alter management in the acute or long-term setting. Consequently, some guidelines explicitly recommend not using the presence of DVT as a criterion to guide anticoagulation duration in PE [10], and currently, the presence of DVT in someone already diagnosed with PE does not alter the choice or duration of anticoagulation.

CUS might play a role in cases of confirmed PE, when anticoagulation is contraindicated and placement of an inferior vena cava filter is considered [20, 21]. In our cohort, 16 out of 21 patients who received an inferior vena cava filter underwent CUS, of which 13 were positive. CUS is also used in cases where there is uncertainty about the need for anticoagulation, such as in the case of an isolated subsegmental filling defect on CTPA. Guidelines recommend the use of additional imaging studies, including alternative chest imaging or CUS, to confirm or exclude VTE in these cases in patients without active malignancy [9]. There was documented uncertainty of the PE diagnosis in 21 patients based on chest imaging, of which nine patients had CUS performed.

Overall, CUS use in PE should be limited to specific situations; within our study, we did not observe these in most patients who underwent CUS. We did note a large variation between individual physicians in the rates of CUS use, which may indicate physician preference as guiding its use.

### 4.2. Troponin and TTE

Investigation of the right ventricle is traditionally of great interest in the diagnostic and management pathway of PE, given PE-related death is primarily due to the right ventricular failure. Measurements of the right ventricle, in the form of imaging (TTE/CTPA) and/or cardiac biomarkers (high-sensitivity troponin or brain natriuretic peptide), aid the physician in stratifying the severity of PE in conjunction with risk-stratifying tools such as the PE severity index (PESI) [9, 22]. Right ventricle to left ventricle diameter measurements on CTPA may also be useful for prediction of cardiac dysfunction. Both troponin investigation and TTE give incremental prognostic information, with the combination of both tests yielding the best result in a Swiss study [23]. Differentiating between high-, intermediate-, and low-risk PE may assist in decision-making about the level of monitoring required [9, 10, 12]. Over one-third of patients in our study did not undergo dedicated (additional to the diagnostic test) investigation of the function of the right ventricle. A significant portion of patients underwent additional imaging with TTE despite diagnosis on CTPA (and presumed ability to determine RV to LV diameter ratio).

Guideline recommendations vary and are summarized in Table 4. ESC/ERS guidelines [9] recommend evaluating the right ventricular dysfunction in all patients, regardless of clinical indicators (Class IIa recommendation, Level B evidence), based on a 2019 systematic review and meta-analysis [24], but other guidelines recommend against routine evaluation due to conflicting evidence and instead recommend their use in circumstances when they may influence the patient's trajectory, e.g., choice of intensive care or general ward-based monitoring [12]. The evidence underpinning the use of measures of the right ventricular dysfunction is conflicting as to their ability to predict mortality or lead to a meaningful change in the patient's clinical outcome [24–26]. Hence, the decision of whether to perform dedicated investigation of the right ventricle is largely left to the individual physician, leading to the clinical variation seen. Anecdotally, patients at perceived high risk of deterioration are more likely to have these performed; this was reflected in our study, with troponins performed in all patients with proximal PE on CTPA, but in only 43% of patients with distal PE.

### 4.3. Follow-Up Reimaging

Specialist follow-up is recommended in all patients with acute PE, due to the small but significant risk of CTEPH [9]. Up to 50% of patients with acute PE experience the poorly understood “post-PE syndrome” (persistent dyspnea, exercise limitation, and impaired quality of life that persist for longer than 3 months after effective anticoagulation for acute PE) [3]; most of these patients do not have CTEPH. Most PEs resolve rapidly following commencement of anticoagulation [27]; however, in some patients, there are persistent abnormalities on imaging, which may be in the form of filling defects (CTPA) or mismatch defects (V-Q scan). Patients with persistent symptoms, particularly dyspnea, at the 3-month mark may suffer from pulmonary hypertension and should undergo TTE as their first postacute phase investigation (Class IIa recommendation, Level C evidence) [9, 28].

There is little evidence to guide physicians' management of asymptomatic persistent abnormalities on imaging studies after completion of treatment. Routine use of reimaging for PE in all patients is not recommended, as studies have shown little correlation between symptoms and residual defects on imaging at the 3- or 6-month mark [29]. Extension of anticoagulation is recommended based on risk factors for recurrent VTE and not persistent imaging abnormalities [9].

In our study, clinicians organized routine follow-up imaging in 12% of patients, of which the majority were in Group A. In all cases, the repeat imaging ordered was the original scan upon which the diagnosis of PE was made (i.e., CTPA or V-Q); none of the physicians ordered TTE for routine follow-up imaging. Anecdotally, some physicians recommend follow-up imaging at the completion of treatment, to have a baseline to which further scans can then be compared in case of recurrent VTE. This practice is based on some limited evidence, although there is paucity of long-term studies showing a benefit [30].

### 4.4. Study Limitations

This study was a single-center study, and the results may not be representative of other settings. Given that the evidence underpinning many guideline recommendations for the management of PE is weak, it is, however, reasonable to assume that clinical variation in the management of PE is a widespread issue. Robust studies are required to address some of the knowledge gaps leading to variation in clinical practice.

In conclusion, we have demonstrated substantial variation in diagnostic and treatment decisions.

## 5. Conclusion

Our study found substantial variation related to the management pathway of PE, namely, the use of CUS in already diagnosed PE, right ventricular investigations (TTE or biomarkers) for prognostication for PE, and follow-up imaging to detect residual PE. Where guideline recommendations exist for these diagnostic tests, the underpinning evidence is generally weak or conflicting, and robust research studies are therefore needed to address these evidence gaps.

## Figures and Tables

**Table 1:** Patient characteristics.

**Patient characteristics**	**n** ** (%), mean (SD)**
Age	
Years, mean (SD)	62.3 (17.4)
Sex	
Male	159 (53%)
Female	141 (47%)
Length of stay (days)	
1–2	56 (19%)
3–4	44 (15%)
5–7	46 (15%)
8+	154 (51%)
Presenting complaint	
Chest pain	94 (31%)
Dyspnea	88 (29%)
Syncope	19 (6%)
Other	99 (33%)
Risk factors for VTE (more than one possible)	
Nil	67 (22%)
Previous VTE	59 (20%)
Pregnancy	6 (2%)
Hormonal therapy (contraceptive pill or hormone replacement therapy)	11 (4%)
Malignancy	100 (33%)
Recent travel	19 (6%)
Recent surgery	49 (16%)
Recent admission	48 (16%)
Renal disease (nephrotic syndrome)	13 (4%)
Inherited thrombophilia	4 (1%)
Trauma	18 (6%)
Smoking	29 (10%)
Preexisting antithrombotics/anticoagulants	
Nil	200 (67%)
Antithrombotic	
Single	50 (17%)
Dual	10 (3%)
Anticoagulant	
Warfarin	9 (3%)
Direct-acting agent	19 (6%)
Other	11 (4%)
Dual antithrombotics + anticoagulation (“triple therapy”)	1 (1%)
Renal function	
Estimated glomerular filtration rate < 30 mL/min/1.73m^2^	34 (11%)
Estimated glomerular filtration rate > 30 mL/min/1.73m^2^	266 (89%)

**Table 2:** Diagnostic pathways and investigations.

**Total diagnosed**	**All ** **patients**	**Location of initial ** **presumed diagnosis of ** **pulmonary embolism**			**Treating physician**		
		**Emergency ** **department**	**Inpatient ** **(ICU/ward)**		**Group A + B ** **(respiratory)**	**Group C ** **(nonrespiratory)**	
	**n** ** (%)**	**n** ** (%)**	**n** ** (%)**	**p** ** value**	**n** ** (%)**	**n** ** (%)**	**p** ** value**
	300	161 (54)	139 (46)	–	204 (68)	96 (32)	–
Initial investigation							
CT pulmonary angiogram	148 (49)	84 (52)	64 (46)	< 0.0001	105 (51)	43 (45)	0.003
Ventilation-perfusion imaging	43 (14)	19 (12)	24 (17)	–	23 (11)	20 (21)	–
D-dimer	40 (13)	33 (21)	7 (5)	–	36 (18)	4 (4)	–
Lower limb compression ultrasonography	11 (4)	7 (4)	4 (3)	–	8 (4)	3 (3)	–
Transthoracic echocardiogram	4 (1)	0 (0)	4 (3)	–	3 (1)	1(1)	–
Incidental CT scan	53 (18)	17 (11)	36 (26)	–	28 (14)	25 (26)	–
Chest x-ray	1 (0.3)	1 (0.6)	0 (0)	–	1 (0.5)	0 (0)	–
Confirmatory diagnostic modality							
CT pulmonary angiogram	199 (66)	124 (77)	75 (54)	< 0.0001	147 (72)	52 (54)	0.004
Ventilation-perfusion imaging	60 (20)	30 (19)	30 (22)	–	37 (18)	23 (24)	–
Incidental CT scan	41 (14)	7 (4)	34 (24)	–	20 (10)	21 (22)	–
Ancillary tests performed							
Lower limb doppler	132 (44)	66 (41)	66 (47)	0.26	100 (49)	32 (33)	0.01
Positive	69 (52)	37 (56)	32 (48)	0.057	53 (53)	16 (50)	0.45
Negative	59 (45)	25 (38)	34 (52)	–	43 (43)	16 (50)	–
Pending on discharge (outpatient scan)	4 (3)	4 (6)	0 (0)	–	4 (4)	0 (0)	–
Troponin	167 (56)	109 (68)	58 (42)	< 0.0001	133 (65)	34 (36)	< 0.0001
Echocardiogram	82 (27)	29 (18)	53 (38)	< 0.0001	59 (29)	23 (24)	0.37
Thrombophilia screening	43 (14)	27 (17)	16 (12)	0.19	36 (18)	7 (7)	0.02
Positive	6 (14)	3 (11)	3 (19)	0.48	5 (14)	1 (13)	0.90

**Table 3:** Management and follow-up plans.

**Management pathways**	**All patients**	**Treating physician**		
		**Group A + B (respiratory)**	**Group C (nonrespiratory)**	
	**n** ** (%)**	**n** ** (%)**	**n** ** (%)**	**p** ** value**
Thrombolysis given	9 (3%)	8 (3%)	1 (1%)	–
Initial anticoagulant choice				
Low molecular weight heparin	138 (46)	92 (45)	46 (48)	0.17
Direct-acting anticoagulant	52 (17)	37 (18)	15 (16)	–
Heparin	99 (33)	71 (35)	28 (29)	–
Warfarin	4 (1)	1 (0.5)	3 (3)	–
Nil	7 (2)	3 (1)	4 (4)	–
Long-term anticoagulant choice				
Low molecular weight heparin	51 (17)	21 (10)	30 (31)	< 0.0001
Direct-acting anticoagulant	162 (54)	128 (63)	34 (35)	–
Warfarin	63 (21)	45 (22)	18 (19)	–
Nil	24 (8)	10 (5)	14 (15)	–
Duration of intended treatment				
3 months	29 (10)	27 (13)	2 (2)	0.0008
6 months	52 (17)	41 (20)	11 (11)	–
Lifelong	44 (15)	31 (15)	13 (14)	–
To be determined	153 (51)	95 (47)	58 (60)	–
Nil treatment	22 (7)	10 (5)	12 (13)	–
Re-imaging planned				
No (or not documented)	264 (88)	171 (84)	93 (97)	0.0012
Yes	36 (12)	33 (16)	3 (3)	–
CT pulmonary angiogram	26 (9)	25 (76)	1 (33)	0.051
Ventilation-perfusion imaging	6 (2)	4 (12)	2 (67)	–
Lower limb doppler	4 (1)	4 (12)	0 (0)	–
Follow-up planned				
Nil	83 (28)	44 (22)	39 (41)	< 0.0001
3 months	137 (46)	116 (57)	21 (22)	–
6 months	22 (7)	17 (8)	5 (5)	–
Other	58 (19)	27 (13)	31 (33)	–
Outcome				
Discharge	278 (93)	198 (97)	80 (83)	< 0.0001
Death in hospital	21 (7)	6 (3)	15 (16)	–
Discharge against medical advice	1 (1)	0 (0)	1 (1)	–

**Table 4:** Clinical questions concerning diagnostic investigations in PE and their recommendations within PE guidelines.

**Clinical question**	**ESC/ERS (2017) [** 9 **]**	**ASH (2020) [** 10 **]**	**THANZ (2019) [** 11 **]**	**Chest (2016) [** 12 **]**
Performing CUS in patients with diagnosed PE	Not addressed	Not addressed	Not addressed	Not addressed
Performing investigation for right ventricular strain in patients with diagnosed PE	Considered in all patients (Class IIa recommendation, Level B evidence)	Not addressed	Not addressed	Patients in whom the level of monitoring or need for thrombolysis is unclear
Follow-up reimaging at specialist review	If symptoms persist only (Class IIa recommendation, Level C evidence)	Not addressed	If symptoms persist only	Not addressed

**Table 5:** Collected variables.

**Category**	**Variable**
Demographic and encounter details	Medical record number
	Sex
	Age
	Length of stay

Presentation characteristics	Presenting complaint
	Location of initial suspicion for pulmonary embolism
	Preexisting antithrombotics/anticoagulants and their indication
	Renal function

Risk factors	Previous venous thromboembolism
	Pregnancy
	Hormonal therapy (contraceptive pill or hormone replacement therapy)
	Malignancy
	Recent travel
	Recent surgery
	Recent admission
	Renal disease (nephrotic syndrome)
	Inherited thrombophilia
	Trauma
	Smoking

Diagnostic pathway	Wells' score and result
	Initial and subsequent investigations
	D-dimer
	CT pulmonary angiogram or incidental CT scanning
	Ventilation-perfusion imaging
	Transthoracic echocardiogram
	Troponin
	Lower limb doppler
	Thrombophilia screen
	Confirmatory diagnostic modality

Management pathway	Thrombolysis
	Anticoagulation
	Initial choice
	Long-term choice and reason if not direct acting anticoagulant
	Intended duration of treatment
	Inferior vena cava filter use
	Reimaging if planned
	Follow-up duration

Decision-maker	Respiratory physician if involved, or other teams

## Data Availability

All data is available by contacting the corresponding author at devesh.thakkar@health.nsw.gov.au.
